# Competitive exclusion of Salmonella enteritidis by Salmonella gallinarum in poultry.

**DOI:** 10.3201/eid0605.000501

**Published:** 2000

**Authors:** W Rabsch, B M Hargis, R M Tsolis, R A Kingsley, K H Hinz, H Tschäpe, A J Bäumler

**Affiliations:** Robert Koch Institute, Wernigerode, Germany.

## Abstract

Salmonella Enteritidis emerged as a major egg-associated pathogen in the late 20th century. Epidemiologic data from England, Wales, and the United States indicate that S. Enteritidis filled the ecologic niche vacated by eradication of S. Gallinarum from poultry, leading to an epidemic increase in human infections. We tested this hypothesis by retrospective analysis of epidemiologic surveys in Germany and demonstrated that the number of human S. Enteritidis cases is inversely related to the prevalence of S. Gallinarum in poultry. Mathematical models combining epidemiology with population biology suggest that S. Gallinarum competitively excluded S. Enteritidis from poultry flocks early in the 20th century.


Perspective

### Competitive Exclusion of *Salmonella* Enteritidis by *Salmonella* Gallinarum in Poultry

**Wolfgang Rabsch,* Billy M. Hargis,† Renée M. Tsolis,† Robert A. Kingsley,† Karl-Heinz Hinz,‡ Helmut Tschäpe,* and Andreas J. Bäumler†**

*Robert Koch Institute, Wernigerode, Germany; †Texas A&M University, College Station, Texas, USA; ‡School of Veterinary Medicine, Hanover, Germany

*Salmonella* Enteritidis emerged as a major egg-associated pathogen in the late 20th century. Epidemiologic data from England, Wales, and the United States indicate that *S.* Enteritidis filled the ecologic niche vacated by eradication of *S.* Gallinarum from poultry, leading to an epidemic increase in human infections. We tested this hypothesis by retrospective analysis of epidemiologic surveys in Germany and demonstrated that the number of human *S.* Enteritidis cases is inversely related to the prevalence of *S.* Gallinarum in poultry. Mathematical models combining epidemiology with population biology suggest that *S.* Gallinarum competitively excluded *S.* Enteritidis from poultry flocks early in the 20th century.

The avian-adapted serovar *Salmonella* Gallinarum, which includes two biovars, Gallinarum and Pullorum, was endemic in poultry flocks in Europe and the Americas in the early 20th century (1). To reduce economic losses to the poultry industry, national surveillance programs were established in the United States (National Poultry Improvement Plan, 1935) and England and Wales (Poultry Stock Improvement Plan, 1939). Since *S.* Gallinarum (antigen formula O9,12:-:-) has no animal reservoir other than domestic and aquatic fowl, the test-and-slaughter method of disease control under these surveillance programs led to its eradication from commercial poultry flocks in the United States, England, and Wales by the 1970s (1,2). At that time, the number of human cases of infection with serovar *S.* Enteritidis (antigen formula O9,12:g,m:1,7) began to increase in these countries (3,4). By the 1980s, *S.* Enteritidis had emerged as a major concern for food safety in Europe and the Americas (5); by 1990 it was the most frequently reported *Salmonella* serovar in the United States (6). Most *S.* Enteritidis outbreaks in Europe and the United States are associated with foods containing undercooked eggs (7-10). Eggs can become contaminated with *S.* Enteritidis through cracks in the shell after contact with chicken feces or by transovarian infection (11). Thus, laying hens were the likely source of the *S.* Enteritidis epidemic in Europe and the Americas.

The inverse relationship between the incidence of *S.* Gallinarum infection in chickens and egg-associated *S.* Enteritidis infections in humans prompted the hypothesis that *S.* Enteritidis filled the ecologic niche vacated by eradication of *S.* Gallinarum from domestic fowl (12). The hypothesis suggests that the epidemic increase in human *S.* Enteritidis cases in several geographic areas can be traced to the same origin, accounting for the simultaneous emergence of *S.* Enteritidis as a major egg-associated pathogen on three continents (5). A connection between the epidemics in Western Europe and the United States was not apparent from analysis of epidemic isolates. Although most human cases from England and Wales result from infection with *S.* Enteritidis phage type 4 (PT4), most cases in the United States are due to infections with PT8 and PT13a (13,14). The PT4 clone is genetically distinct from PT8 and 13a, as shown

Address for correspondence: Andreas J. Bäumler, Department of Medical Microbiology and Immunology, Texas A&M University, 407 Reynolds Medical Building, College Station, TX 77843-1114; fax: (979) 845-3479; e-mail: baumler@tamu.edu.

by IS200 profiling, ribotyping, and restriction fragment length polymorphism of genomic DNA fragments separated by pulsed-field gel electrophoresis (15). The reasons for the differing clonal isolates in the United States and Western Europe are unknown. *S*. Enteritidis was likely introduced into poultry flocks from its rodent reservoir (12). The geographic differences in predominant phage types may reflect the fact that at the time of introduction into poultry flocks, different *S*. Enteritidis strains were endemic in rodent populations in Europe and the United States. Subsequently, *S*. Enteritidis strains with the highest transmissibility may have become predominant in poultry flocks on each continent. An alternative explanation for the predominance of PT4 in England and Wales is its introduction into poultry breeding lines in the early 1980s (16), which may have accelerated the epidemic spread of PT4 in laying hens and resulted in its dominance in human isolates from England and Wales. However, factors responsible for the beginning of the *S*. Enteritidis epidemic should be considered separately from those important for its subsequent spread within the poultry industry. These factors were not specific to PT4 but rather allowed different phage types to emerge as egg-associated pathogens on different continents at the same time (5).

One such factor could be the eradication of *S*. Gallinarum from poultry, which would facilitate circulation of *S*. Enteritidis strains within this animal reservoir regardless of phage type. Experimental evidence indicates that immunization with one *Salmonella* serovar can generate cross-immunity against a second serovar if both organisms have the same immunodominant O-antigen on their cell surface (17-19). The immunodominant epitope of the lipopolysaccharide of *S*. Gallinarum and *S*. Enteritidis is the O9-antigen, a tyvelose residue of the O-antigen repeat (20). Immunization of chickens with *S*. Gallinarum protects against colonization with *S*. Enteritidis (21,22) but not *S*. Typhimurium, a serovar expressing a different immunodominant determinant, the O4-antigen (23). Theory indicates that coexistence of *S*. Gallinarum and *S*. Enteritidis in an animal population prompts competition as a result of the shared immunodominant O9-antigen, which generates cross-immunity. Mathematical models predict that the most likely outcome of this competition between serovars is that the serovar with the higher transmission success will competitively exclude the other from the host population (24-26). *S*. Gallinarum may have generated population-wide immunity (flock immunity) against the O9-antigen at the beginning of the 20th century, thereby excluding *S*. Enteritidis strains from circulation in poultry flocks (12). This proposal is based on analysis of epidemiologic data from the United States, England, and Wales. To formally test this hypothesis, we analyzed epidemiologic data from Germany to determine whether the numbers of human *S*. Enteritidis cases are inversely related to those of *S*. Gallinarum cases reported in poultry. We used mathematical models to determine whether our hypothesis is consistent with theoretical considerations regarding transmissibility and flock immunity.

### Inverse Relationship of *S.* Enteritidis and *S.* Gallinarum Isolations in Germany

In West Germany, the number of human *S*. Enteritidis cases was monitored by a national surveillance program (Figure) (Zentrales Überwachungsprogram Salmonella, ZÜPSALM)

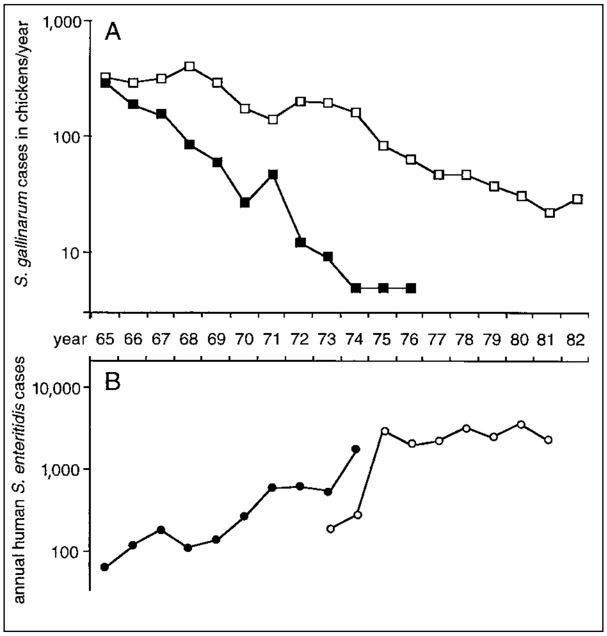

Figure. (A) *S*. Gallinarum infections in chickens in England and Wales (closed squares) (2,27) and the Federal Republic of Germany (open squares) (28). (B) Human cases of *S*. Enteritidis infections per year reported from England and Wales (closed circles) (3,29) and the Federal Republic of Germany (open circles) (Zentrales Überwachungsprogram Salmonella, ZÜPSALM).

from 1973 to 1982. In 1975, the number of human infections began to increase, indicating the beginning of the *S*. Enteritidis epidemic in West Germany. In 1983 the ZÜPSALM program was replaced by a national program for surveillance of foodborne disease outbreaks (Zentrale Erfassung von Ausbrüchen lebensmittelbedingter Infektionen, ZEVALI), implemented by the Department of Public Health (Bundesgesundheitsamt). In the first year of this program, *S*. Enteritidis was responsible for 62 outbreaks, most of which were traced to raw eggs. By 1988, the number of disease outbreaks caused by *S*. Enteritidis had increased to 1,365.

In 1967 in England and Wales, poultry, particularly chickens, became the main human food source of *S*. Enteritidis (3). Before that date, the organism had only sporadically been isolated from poultry (3). A continuous increase in human *S*. Enteritidis cases was recorded from 1968 until the epidemic peaked in 1994 (12,16). Thus, the human *S*. Enteritidis epidemic in England and Wales probably began in 1968 after this organism became associated with a human food source, chickens. The rapid increase in the number of human cases from 1982 to 1988 was probably due to the introduction of PT4 into poultry breeding lines in England and Wales (16). Comparison of data from England and Wales (3,29) showed that *S*. Enteritidis emerged somewhat later in West Germany (Figure).

Eradication of *S*. Gallinarum was among the factors contributing to the emergence of *S*. Enteritidis as a foodborne pathogen (12). To determine whether delayed elimination of avian-adapted *Salmonella* serovars from commercial flocks contributed to the late start of the human epidemic in Germany, we compared the results of surveys performed in poultry flocks in Germany with those from the United Kingdom and the United States. Control programs in the 1930s triggered a steady decline in the incidence of *S*. Gallinarum in poultry flocks in the United States, England, and Wales (1,2,12). By the early 70s, only a few cases of *S*. Gallinarum were reported each year to veterinary investigation centers in England and Wales (27). In Germany, the first national survey performed by the Department of Public Health (Reichsgesundheitsamt) in 1929 showed that 16.3% of birds were seropositive for *S*. Gallinarum (30). Blood-testing performed 20 years later with 6,313 birds in a province (Südbaden) of West Germany still detected 19.5% reactors (31). This high prevalence of *S*. Gallinarum in 1949 likely reflects the fact that after World War II available resources were directed toward rebuilding the poultry industry rather than improving disease control. The comparatively slow decline in the prevalence of *S*. Gallinarum in West Germany is illustrated further by data for cases of disease reported from poultry. The number of *S*. Gallinarum isolations from chicken carcasses received by veterinary laboratories in West Germany was reported by a surveillance program from 1963 to 1981 (28). During this period, the rate of decrease in numbers of *S*. Gallinarum cases in England and Wales was considerably higher than that reported from West Germany (Figure). In each country the numbers of *S*. Gallinarum cases were inversely related to the numbers of human *S*. Enteritidis cases. These data are consistent with the concept that the relative delay in eradicating *S*. Gallinarum from poultry may have contributed to delayed onset of the *S*. Enteritidis epidemic in West Germany.

### Competitive exclusion of *S.* Enteritidis by *S.* Gallinarum

To calculate whether the prevalence of *S*. Gallinarum in chickens was high enough to generate flock immunity against *S*. Enteritidis, we analyzed epidemiologic data by mathematical models combining epidemiology with population biology (24-26). The transmission success of a pathogen is measured by the basic case-reproductive number, $R_0$, which is defined as the average number of secondary cases of infection from a primary case in a susceptible host population (32). In direct transmission, the basic case-reproductive number of a pathogen is directly proportional to the duration, D, for which an infected host can transmit the disease before it is either killed or clear of infection; the probability, ß, by which the disease is transmitted from an infected animal to a susceptible host; and the density of susceptible hosts, X (24).

$$R_0 = ßDX \qquad \text{(equation 1)}$$

After a pathogen is introduced into a susceptible host population, the reproductive rate of the infection declines as a consequence of the removal of a fraction, y, of the susceptible population, X, either by disease-induced death or acquisition of immunity. That is, the effective case-reproductive number, R, will be smaller than the basic case-reproductive number $R_0$.

$$R = ßD (X-Xy) = R_0-R_0y \quad \text{(equation 2)}$$

In an endemic state, each primary case of infection produces, on average, one secondary case. Thus, the effective case-reproductive number in a steady endemic-state situation is R=1. By solving equation 2 for $R_0$, we obtain (33)

$$R_0=1/(1-y) \quad \text{(equation 3)}$$

Since *S*. Gallinarum was endemic in poultry populations at the beginning of the 20th century, its basic case-reproductive number, $R_0$, can be calculated on the basis of epidemiologic data collected before control measures were implemented, by estimating the fraction, y, of birds removed from the susceptible population.

The first method developed for detecting anti-*S*. Gallinarum antibodies was a macroscopic tube agglutination test introduced in 1913 (34). In 1931, the tube agglutination test was partially replaced by the simpler whole-blood test for slide agglutination of stained antigen (35). Initial surveys performed from 1914 to 1929 revealed that on average 9.8% to 23.8% of poultry in Europe and the United States were positive by the tube agglutination test (1,30,36). These data do not provide a direct estimate of the number of immune animals, since both serologic tests are relatively insensitive (37). However, the number of susceptible birds can be estimated by comparing results of serologic surveys with data from vaccination experiments. Immunization with *S*. Gallinarum vaccine strain 9R produces antibody levels high enough to be detected by the whole-blood tube or slide agglutination tests in only a small number of birds (approximately 10%) (20,23). The number of birds protected against challenge with virulent *S*. Gallinarum after a single oral or subcutaneous vaccination is considerably higher (approximately 60%) (23,38). The tube or slide agglutination test results (9.8% and 23.8% of birds, respectively, tested positive) at the beginning of this century suggest that at least 60% were immune to *S*. Gallinarum. In addition to acquired immunity, deaths, which likely occurred in most chicken flocks since *S*. Gallinarum reactors were present on most farms at the time, also reduced the density of susceptible hosts. For instance, only 9 of 144 farms surveyed in Hungary in the 1930s had no *S*. Gallinarum-positive birds (39). The death rates reported from natural outbreaks are 10% to 50%, although higher rates are occasionally reported (40). By the conservative estimate that 90% of birds in a flock will survive an outbreak and approximately 60% of the survivors will have protective immunity, the basic case-reproductive number, $R_0$, of *S*. Gallinarum is estimated to be 2.8.

*S*. Enteritidis does not substantially reduce the density of susceptible animals by causing death. Thus, its basic case-reproductive number can be estimated from the number of birds that remained susceptible during the peak of the *S*. Enteritidis epidemic. Antibody titers in *S*. Enteritidis-infected flocks are generally too low to be detected by the tube or the slide agglutination tests (37,41), presumably because this serovar commonly colonizes birds without causing disease and consequently without triggering a marked immune response. Live attenuated *S*. Enteritidis aroA vaccine does not produce antibody titers detectable by the tube or the slide agglutination tests (42), and oral immunization with this vaccine does not protect against organ colonization with wild-type *S*. Enteritidis (43). Hence, exposure to *S*. Enteritidis does not protect at levels found in birds with previous exposure to *S*. Gallinarum. Indeed, in a survey of flocks naturally infected with *S*. Enteritidis, only one of 114 birds tested strongly positive by the slide agglutination test (37). Experimental evidence indicates that birds exposed to *S*. Gallinarum have strong cross-immunity against colonization with S. Enteritidis. For instance, immunization of chickens with a single dose of *S*. Gallinarum vaccine strain 9R causes similar levels of protection against challenge with *S*. Gallinarum (23,38) and *S*. Enteritidis (22,44). The high degree of cross-immunity suggests that the antibody titers detected by the tube agglutination test are predictive of protection against lethal *S*. Gallinarum infection and of immunity to colonization by *S*. Enteritidis. Applying the criteria used to calculate $R_0$ for *S*. Gallinarum (10% reactors are indicative of 60% protection) to the *S*. Enteritidis data (37) suggests that approximately 5% of birds had protective immunity against this pathogen. From these data, the basic case-reproductive number of *S*. Enteritidis ($R_0$=1.05) is estimated to be considerably lower than that of *S*. Gallinarum.

Several factors should be considered in interpreting these data. Our estimate of the $R_0$ value for *S.* Enteritidis is based on epidemiologic data from the late 1980s. The intensive husbandry of chickens in the latter part of the 20th century has increased the density, X, of susceptible hosts and therefore $R_0$ (equation 1). Furthermore, information on the number of birds in *S.* Enteritidis-infected flocks with positive reactions in the tube agglutination test is sparse, and data from the peak of the epidemic in 1994 are not available. The prevalence of *S.* Enteritidis in poultry has been documented by a survey performed in Lower Saxony, Germany, in 1993, a time when flocks were heavily infected. This study showed that 7.6% of 2,112 laying hens were culture positive at slaughter (45). Although this low prevalence is consistent with a low basic case-reproductive number of *S.* Enteritidis at the peak of the epidemic, these data cannot be used to derive a reliable estimate for the basic case-reproductive number of *S.* Enteritidis at the beginning of the 20th century. Given these limitations, the available epidemiologic evidence appears to be consistent with our hypothesis. From equation 2 ($R=R_0-R_0y$), we estimate that early in the century the number of susceptible birds killed by *S.* Gallinarum (assuming 100% cross-immunity and y = 0.65) reduced the effective case-reproductive number of *S.* Enteritidis to < 1 (R = 0.37). These estimates support the idea that at the beginning of the 20th century *S.* Gallinarum reduced the density of susceptible hosts sufficiently to competitively exclude *S.* Enteritidis from circulation in poultry flocks.

*S.* Enteritidis is unlikely to be eliminated from poultry by relying solely on the test-and-slaughter method of disease control because, unlike *S.* Gallinarum, *S.* Enteritidis can be reintroduced into flocks from its rodent reservoir. Instead, vaccination would be effective in excluding *S.* Enteritidis from domestic fowl because it would eliminate one of the risk factors (loss of flock immunity against the O9-antigen), which likely contributed to the emergence of *S.* Enteritidis as a foodborne pathogen. In fact, much of the decline in human *S.* Enteritidis cases in England and Wales since 1994 has been attributed to the use of an *S.* Enteritidis vaccine in poultry (16). However, serologic evidence that *S.* Gallinarum is more immunogenic than *S.* Enteritidis suggests that a more effective approach for eliciting protection in chickens would be immunization with a live attenuated *S.* Gallinarum vaccine. This approach would restore the natural balance (exclusion of *S.* Enteritidis by a natural competitor) that existed before human intervention strategies were implemented early in the 20th century.

### Acknowledgments

We thank J. Bockemuehl, E. Vielitz, B. Koehler, S. Kautzsch, and H. Meyer for their help with data collection and L.G. Adams and S.M. Townsend for critical comments on the manuscript.

This material is based in part upon work supported by the Texas Advanced Research (Technology) Program under grant number 000089-0051-1999. Work in Dr. Baumler's laboratory is supported by Public Health Service grants AI40124 and AI44170.

Dr. Rabsch is a microbiologist at the National Reference Centre for Salmonella and other enteric infections at the Robert Koch Institute in Wernigerode, Germany. His research involves typing Salmonella isolates for epidemiologic analysis.

### References

1. Bullis KL. The history of avian medicine in the U.S. II. Pullorum disease and fowl typhoid. Avian Dis 1977;21:422-9.
2. Sojka WJ, HI Field. Salmonellosis in England and Wales, 1958-1967. Vet Bull 1970;40:515-31.
3. Lee JA. Recent trends in human salmonellosis in England and Wales: the epidemiology of prevalent serotypes other than *Salmonella* typhimurium. J Hyg (Cambridge) 1974;72:185-95.
4. Aserkoff B, Schroeder SA, Brachman PS. Salmonellosis in the United States—a five-year review. Am J Epidemiol 1970;92:13-24.
5. Rodrigue DC, Tauxe RV, Rowe B. International increase in *Salmonella* enteritidis: a new pandemic? Epidemiol Infect 1990;105:21-7.
6. Mishu B, Koehler J, Lee LA, Rodrigue D, Brenner FH, Blake P, et al. Outbreaks of *Salmonella* enteritidis infections in the United States, 1985-1991. J Infect Dis 1994;169:547-52.
7. St. Louis ME, Morse DL, Potter ME, DeMelfi TM, Guzewich JJ, Tauxe RV, et al. The emergence of grade A eggs as a major source of *Salmonella* Enteritidis infections. New implications for the control of salmonellosis. JAMA 1988;259:2103-7.
8. Henzler DJ, Ebel E, Sanders J, Kradel D, Mason J. *Salmonella* Enteritidis in eggs from commercial chicken layer flocks implicated in human outbreaks. Avian Dis 1994;38:37-43.
9. Coyle E, Palmer S, Ribeiro CD, Jones H, Howard A, Ward L, et al. *Salmonella* enteritidis phage type 4 infection: association with hen's eggs. Lancet 1988;2:1295-7.
10. Cowden JM, Lynch D, Joseph CA, O'Mahony M, Mawer SL, Rowe B, et al. Case-control study of infections with *Salmonella* enteritidis phage type 4 in England. Br Med J 1989;299:771-3.

11. Snoeyenbos GH, Smyser CF, Van Roekel H. *Salmonella* infections of the ovary and peritoneum of chickens. Avian Dis 1969;13:668-70.
12. Bäumler AJ, Hargis BM, Tsolis RM. Tracing the origins of *Salmonella* outbreaks. Science 2000;287:50-2.
13. Hickman-Brenner FW, Stubbs AD, Farmer JJ. Phage typing of *Salmonella* enteritidis in the United States. J Clin Microbiol 1991;29:2817-23.
14. Wall PG, Ward LR. Epidemiology of *Salmonella* enterica serovar Enteritidis phage type 4 in England and Wales. In: Saeed AM, Gast RK, Potter ME, Wall PG, editors. *Salmonella* enterica serovar Enteritidis in humans and animals. Ames (IA): Iowa State University Press; 1999. p. 19-25.
15. Olsen JE, Skov MN, Threlfall EJ, Brown DJ. Clonal lines of *Salmonella* enterica serotype Enteritidis documented by IS200-, ribo-, pulsed-field gel electrophoresis and RFLP typing. J Med Microbiol 1994;40:15-22.
16. Ward LR, Threlfall J, Smith HR, O'Brien SJ. *Salmonella* Enteritidis epidemic [letter; comment]. Science 2000;287:1753-4; discussion 1755-6.
17. Collins FM, Mackaness GB, Blanden RV. Infection-immunity in experimental salmonellosis. J Exp Med 1966;124:601-19.
18. Lyman MB, Stocker BA, Roantree RJ. Evaluation of the immune response directed against the *Salmonella* antigenic factors O4,5 and O9. Infect Immun 1979;26:956-65.
19. Hormaeche CE, Mastroeni P, Harrison JA, Demarco de Hormaeche R, Svenson S, Stocker BA. Protection against oral challenge three months after i.v. immunization of BALB/c mice with live Aro *Salmonella* Typhimurium and *Salmonella* Enteritidis vaccines is serotype (species)-dependent and only partially determined by the main LPS O antigen. Vaccine 1996;14:251-9.
20. Barrow PA, Berchieri A Jr, al-Haddad O. Serological response of chickens to infection with *Salmonella* gallinarum-S. pullorum detected by enzyme-linked immunosorbent assay. Avian Dis 1992;36:227-36.
21. Nassar TJ, al-Nakhli HM, al-Ogaily ZH. Use of live and inactivated *Salmonella* enteritidis phage type 4 vaccines to immunise laying hens against experimental infection. Rev Sci Tech 1994;13:855-67.
22. Sterner F, Hein R. An attenuated *Salmonella* gallinarum live vaccine induces long-term protection against *Salmonella* enteritidis challenge in chickens. Presented at the International Symposium on Food-Borne Salmonella in Poultry; 1998; Baltimore, MD.
23. Silva EN, Snoeyenbos GH, Weinack OM, Smyser CF. Studies on the use of 9R strain of *Salmonella* gallinarum as a vaccine in chickens. Avian Dis 1981;25:38-52.
24. Anderson RM. Evolutionary pressures in the spread and persistence of infectious agents in vertebrate populations. Parasitology 1995;111(Suppl):S15-31.
25. Gupta S, Swinton J, Anderson RM. Theoretical studies of the effects of heterogeneity in the parasite population on the transmission dynamics of malaria. Proc R Soc Lond B Biol Sci 1994;256:231-8.
26. Gupta S, Maiden MC, Feavers IM, Nee S, May RM, Anderson RM. The maintenance of strain structure in populations of recombining infectious agents. Nat Med 1996; 2:437-42.
27. Sojka WJ, Wray C, Shreeve J, Benson AJ. Incidence of salmonella infection in animals in England and Wales 1968-1974. J Hyg (Lond) 1977;78:43-56.
28. Pietzsch O. Salmonellose-Überwachung bei Tieren und Lebensmitteln in der Bundesrepublik Deutschland. Zeitraum 1963-1981. Bundesgesundheitsblatt und Vet Med Hefte 1985, report no. 3.
29. McCoy JH. Trends in salmonella food poisoning in England and Wales, 1941-72. J Hyg (Lond) 1975;74:271-82.
30. Beller K, Zunker M. Sammelbericht über die mit Mitteln des Reichs und Preußischen Ministeriums für Ernährung und Landwirtschaft durchgeführte Forschungsarbeit auf dem Gebiete der Geflügelkrankheiten. Reichsgesundheitsamt. 1936.
31. Nassal J. Bekämpfung der Pullorum-Infektion im Ramen des Geflügel-Gesundheitsdienstes in Südbaden von 1949 bis 1955. Berl Munch Tierarztl Wochenschr 1957;70:24-8.
32. Anderson RM, May RM. Coevolution of host and parasites. Parasitology 1982;85:411-26.
33. Anderson RM, May RM. Immunisation and herd immunity. Lancet 1990;335:641-5.
34. Jones FS. The value of the macroscopic agglutination test in detecting fowls that are harboring Bact. pullorum. J Med Res 1913;27:481-95.
35. Schaffer JM, MacDonald AD. A stained antigen for the rapid whole blood test for pullorum disease. J Am Vet Med Assoc 1931;32:236-40.
36. Wilson JE. Pullorum disease in Scotland, 1926-1966. Br Vet J 1967;123:139-44.
37. Cooper GL, Nicholas RA, Bracewell CD. Serological and bacteriological investigations of chickens from flocks naturally infected with *Salmonella* enteritidis. Vet Rec 1989;125:567-72.
38. Bouzoubaa K, Nagaraja KV, Kabbaj KZ, Newman JA, Pomeroy BS. Feasibility of using proteins from *Salmonella* gallinarum vs. 9R live vaccine for the prevention of fowl typhoid in chickens. Avian Dis 1989;33:385-91.
39. Grzimek B. Krankes Geflügel, 4th ed. Berlin: Verlag Fritz Pfenningsdorff; 1943. p. 16-34.
40. Hall WJ, Legenhausen DH, MacDonald AD. Studies of fowl typhoid. I. Nature and dissemination. Poultry Science 1949; 28:344-62.
41. Barrow PA. ELISAs and the serological analysis of *Salmonella* infections in poultry: a review. Epidemiol Infect 1992; 109:361-9.
42. Cooper GL, Venables LM, Nicholas RA, Cullen GA, Hormaeche CE. Vaccination of chickens with chicken-derived *Salmonella* Enteritidis phage type 4 aroA live oral *Salmonella* vaccines. Vaccine 1992;10:247-54.
43. Cooper GL, Venables LM, Woodward MJ, Hormaeche CE. Vaccination of chickens with strain CVL30, a genetically defined *Salmonella* Enteritidis aroA live oral vaccine candidate. Infect Immun 1994;62:4747-54.
44. Witvliet M, Vostermans T, van den Bosch J, de Vries TS, Pennings A. Induction of cross-protection against *Salmonella* enteritidis by the *Salmonella* gallinarum 9R vaccine. Presented at the International Symposium on Food-Borne Salmonella in Poultry; 1998; Baltimore, Maryland.
45. Hinz KH, Legutko P, Schroeter A, Lehmacher W, Hartung M. Prevalence of motile salmonellae in egg-laying hens at the end of the laying period. Zentralbl Veterinarmed [B]. 1996; 43:23-33.
